# Refraction and How to Refract, Including Sections on Optics, Retinoscopy, the Fitting of Spectacles and Eye-Glasses, Etc.

**Published:** 1900-05

**Authors:** 


REFRACTION AND HOW TO REFRACT, Including Sections on Optics, Retinoscopy, The Fitting of Spectacles and Eye-glasses, Etc. By JAMES THORINGTON, A. M., M. D., Adjunct Professor of Ophthalmology in the Philadelphia Polyclinic and College for Graduates in Medicine; Assistant Surgeon at Wills' Eye Hospital; Associate Member of the American Ophthalmological Society; Fellow of the College of Physicians of Philadelphia; Member of the American Medical Association; Ophthalmologist to the Elwyn and the Vineland Training Schools for Feeble-minded Children; Resident Physician and Surgeon Panama Railroad Company at Colon (Aspinwall), Isthmus of Panama, 1882–1889, etc. Two hundred illustrations, thirteen of which are colored. Philadelphia: P. Blakiston's Son & Co., 1012 Walnut Street. 1900.

"Refraction and how to refract" is essentially a book for beginners in ophthalmology. Much of the subject-matter may be found in the text-books on diseases of the eye, but there are also many definitions and explanations, many suggestions as to the methods of examination, many hints as to correction of refractive errors that are not found there. The subjects are concisely handled, and the author succeeds in making himself clearly understood, both in the didactic part of the work, and in the illustrative cases which he has incorporated. He very properly advises the use of cycloplegics in testing for refractive errors, except in presbyopes and in cases where it is impracticable or improper to use them. He emphasizes retinoscopy, and justly so, as the most reliable objective method of determining the refraction of the eye, and gives full directions as to how to use it.

Besides treating of refraction and accommodation of the eye and the methods and instruments applicable to their determination, he also gives a brief chapter on the ocular muscles and one on the adjustment of eye-glasses and spectacles. The book, in short, is what it claims to be, an elementary work on refraction and the optical correction of refractive errors. It is a good book for a beginner, and will serve as a helpful introduction to that deeper study of the subject which is so necessary to good ophthalmological work. A. A. H.

